# Biotransfer of β-*N*-Methylamino-l-alanine (BMAA) in a Eutrophicated Freshwater Lake

**DOI:** 10.3390/md13031185

**Published:** 2015-03-02

**Authors:** Sandra Lage, Heléne Annadotter, Ulla Rasmussen, Sara Rydberg

**Affiliations:** 1 Department of Ecology, Environment and Plant Sciences, Stockholm University, 10654 Stockholm, Sweden; E-Mails: sandra.lage@su.se (S.L.); ulla.rasmussen@su.se (U.R.); 2 Regito AB, 28022 Vittsjö, Sweden; E-Mail: ha@regito.com

**Keywords:** Lake Finjasjön, β-*N*-methylamino-l-alanine, bioaccumulation, phytoplankton, fish

## Abstract

β-*N*-Methylamino-l-alanine (BMAA), a neurotoxic non-protein amino acid, plays a significant role as an environmental risk factor in neurodegenerative diseases, such as amyotrophic lateral sclerosis. BMAA producers occur globally, colonizing almost all habitats and represent species from distinct phytoplanktonic groups, *i.e.*, cyanobacteria, diatoms, and dinoflagellates. Bioaccumulation of BMAA in invertebrate and vertebrate organisms has also been registered around the globe. In the Baltic Sea, BMAA has been detected in several commercial fish species, raising the question of the bioaccumulation of BMAA in Swedish limnic systems. Here we find the presence of BMAA in water samples from Lake Finjasjön and identify its bioaccumulation patterns in both plankti-benthivorous and piscivorous fish, according to fish species, total weight, gender, and season of collection. For the first time, a large number of fish individuals were used in order to draw conclusions on BMAA bioaccumulation in a closed ecological community based on a statistical approach. We may, therefore, conclude that feeding patterns (plankti-benthivorous) and increased age of fish may lead to a higher tissue concentration of BMAA.

## 1. Introduction

β-Methylamino-l-alanine (BMAA), a non-protein amino acid, was first isolated from the seeds of *Cycas circinalis*, currently known as *Cycas micronesica* Hill, in Guam in the Western Pacific Ocean [1,2]. There, BMAA was linked to the high rate of amyotrophic lateral sclerosis/parkinsonism-dementia complex (ALS-PDC) among the indigenous people of this island [3,4]. In 2003, BMAA was found to be produced by the cyanobacterium *Nostoc* sp. living symbiotically with the coralloid roots of the cycad trees [5]. There are several potential mechanisms by which BMAA may cause neurological injury [6,7,8] and environmental exposures may contribute to the development of neurodegenerative disorders [9,10].

BMAA is produced by species representative of cyanobacteria, diatoms, and dinoflagellates [11,12,13]. These species are globally distributed and present in terrestrial, brackish, freshwater, and marine habitats. BMAA has been detected in desert dust in the Persian Gulf [14], several water bodies, including urban waters in the Netherlands [15], 11 freshwater lakes and one brackish water body in Britain [16], cyanobacteria isolates from South African freshwater impoundments [17], springs from a Gobi Desert oasis [18], and marine and freshwater ecosystems in China [19]. Phytoplankton surface blooms are also a recurring phenomenon in the Baltic Sea [20,21]. In the summer months (June–August), these blooms are dominated by the cyanobacterial genera *Nodularia* and *Aphanizomenon*, which have also been demonstrated to produce BMAA [11,22,23]. In the Baltic Sea, larger blooms of diatoms are present in spring (March–May). Jonasson *et al.* [23] found that zooplankton, which naturally feed on cyanobacteria, contain clearly higher levels of BMAA than do the BMAA producers. In addition, several fish tissues were found to contain up to 200 times higher concentrations of BMAA than did the cyanobacteria. The results obtained in this Baltic Sea study revealed, for the first time, that BMAA was biotransferred in an aquatic ecosystem outside Guam.

The Baltic Sea is not the only source of commercial fish in Sweden, a country containing many lakes, including some of the biggest in Europe. In 2011, the four biggest Swedish lakes, *i.e.*, lakes Mälaren, Hjälmaren, Vättern, and Vänern, together contributed 1484 metric tons of commercial fish. This can be compared with the 130,000 metric tons of fish caught in the Baltic Sea, approximately 90,000 tons of which were used as fodder-fish [24]. The large amount of commercial fish used for human consumption from freshwater lakes is of major concern, as Swedish society might be exposed to BMAA through such fish consumption.

To infer any possible transfer of BMAA within Swedish limnic ecosystems, Lake Finjasjön was selected as a eutrophicated model lake. The small size of the lake favors detailed research into the several trophic levels and their interrelationships, therefore, providing a greater understanding of BMAA bioaccumulation patterns. This shallow lake has been suffering from eutrophication since the early 20th century and consequently is occasionally affected by major blooms of toxic cyanobacterial species (*i.e.*, *Microcystis*) [25]. Over the years, several restoration schemes were unsuccessfully implemented, until 1992 when a top-down control strategy restored the native fauna and flora composition, leading to reduced microcystin-producing cyanobacteria [25]. Lake Finjasjön currently has a healthy balance between cyanobacteria and diatoms year round, with increased surface blooms of cyanobacteria between July and September. Although the microcystin-producing cyanobacteria are no longer a problem, several of the cyanobacterial species (*i.e.*, *Aphanizomenon klebahnii*, *Microcystis aeruginosa*, and *Anabaena* sp.) included in the phytoplanktonic composition in Finjasjön are reportedly BMAA-producing species [11,26].

Noteworthy is that BMAA can potentiate neuronal injury induced by other neurotoxic agents, even if both compounds alone have non-toxic concentrations [7,27]. One of these neurotoxic agents is mercury, which will be analyzed together with BMAA.

The present study aims to confirm BMAA production by the phytoplanktonic community (*i.e.*, water samples) and the transfer of BMAA to several fish species at different trophic levels in Lake Finjasjön. Considering the several reports of BMAA occurrence in aquatic systems [23,28,29,30] it is of interest to clarify BMAA bioaccumulation trends in the different fish tissues and fish species through a systematic study with a high number of individuals, which is the main purpose of this study.

## 2. Results

BMAA was detected in three of the four water samples from Lake Finjasjön (Table 1). All positive samples had similar total (*i.e.*, free plus protein-associated) BMAA concentrations.

**Table 1:** BMAA levels in water samples collected in Lake Finjasjön, April 2012.

	BMAA, µg·g^−1^ DW ± SD *
Sample 1	0.002 ± 0.001
Sample 2	0.006 ± 0.002
Sample 3	ND
Sample 4	0.004 ± 0.001

ND = not detected; * Mean BMAA concentrations ± Standard deviation, *n* = 3 analytical replicates.

**Figure 1:**
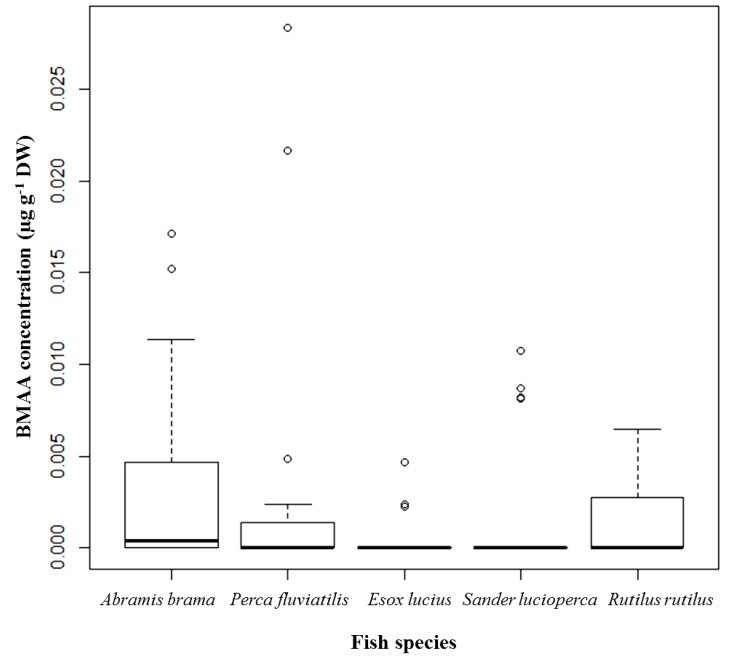
BMAA concentrations detected and quantified in brain tissue of selected fish species from Lake Finjasjön; distribution by species: *Abramis brama* (Bream), *n* = 32; *Perca fluviatilis* (Perch), *n* = 29; *Esox lucius* (Pike), *n* = 22; *Sander lucioperca* (Pike-perch), *n* = 29; and *Rutilus rutilus* (Roach), *n* = 24; median, 75th quartile, maximum, and outliers.

Fish tissues (*i.e.*, brain, muscle, liver, and kidney) were screened for BMAA using UPLC-MS/MS. Quantifiable BMAA peaks were found in 16 of 32 (50%) brain samples from *Abramis brama*, in 8 of 29 (28%) brain samples from *Perca fluviatilis*, in 3 of 22 (14%) brain samples from *Esox lucius*, in 4 of 29 (14%) brain samples from *Sander lucioperca*, and in 9 of 24 (38%) brain samples from *Rutilus rutilus* (Table 2). A relatively higher percentage of positive brain samples of *A. brama* and *R. rutilus* were noted in which the BMAA concentrations were 0.0007–0.0152 µg·g^−1^ dry weight (DW) and 0.0010–0.0064 µg·g^−1^ DW, respectively. The highest BMAA concentration, 0.0283 µg·g^−1^ DW (shown as an outlier), was detected in the brain of a female *P. fluviatilis* caught in spring 2012 (Figure 1).

**Table 2:** Positive BMAA results in selected fish species from Lake Finjasjön: *Abramis brama* (Bream), *n* = 32; *Perca fluviatilis* (Perch), *n* = 29; *Esox lucius* (Pike), *n* = 22; *Sander lucioperca* (Pike-perch), *n* = 29; and *Rutilus rutilus* (Roach), per season of collection, gender and fish tissue, *n* of positive samples/total *n*.

	Fall				Spring			
	Female		Male		Female		Male	
	Brain	Muscle	Brain	Muscle	Brain	Muscle	Brain	Muscle
*Abramis brama*	0/0	0/0	3/7	2/7	7/14	4/14	6/11	3/11
*Perca fluviatilis*	2/7	0/7	1/2	1/2	5/20	3/20	0/0	0/0
*Esox lucius*	0/1	0/1	1/6	1/6	1/9	1/9	1/6	0/6
*Sander lucioperca*	0/1	0/1	3/10	3/10	1/16	3/16	0/2	0/2
*Rutilus rutilus*	4/5	0/5	2/4	1/4	3/15	0/15	0/0	0/0

Regarding the dominance of females over males among the fish samples (*i.e.*, female, *n* = 88 *vs.* male, *n* = 48), a comparable percentage of positive brain samples was found for each gender (*i.e.*, 26% and 35% for female and male samples, respectively), which could imply a higher trend for BMAA presence in male brains than in brains dissected from females (Table 2). However, the highest BMAA concentrations were found in female brains.

The seasonal distribution of BMAA-containing brain samples is comparable to the gender distribution. Although the number of fish collected in spring was more than the double the number collected in fall, the percentage of brain samples containing BMAA was 37% in the fall and 26% in the spring (Table 2). This may imply a higher chance of BMAA being present in fish caught in fall, though higher BMAA concentrations were found in fish caught in spring.

From the total of 136 individuals of *A. brama*, *P. fluviatilis*, *E. lucius*, *S. lucioperca*, and *R. rutilus*, only 22 individuals (*i.e.*, 16%) contained quantifiable BMAA in their muscle tissue (Table 2 and Table 3). As observed for the brain samples, the highest percentage of positive BMAA results found in the muscle samples was in *A. brama* individuals. However, no major tendency could be distinguished due to the small number of positive samples.

**Table 3:** BMAA concentrations in quantifiable samples of muscle tissue from selected fish species from Lake Finjasjön: *Abramis brama* (Bream), *n* = 32; *Perca fluviatilis* (Perch), *n* = 29; *Esox lucius* (Pike), *n* = 22; *Sander lucioperca* (Pike-perch), *n* = 29; and *Rutilus rutilus* (Roach), *n* = 24.

Collection Season	Species	BMAA, µg·g^−1^ DW ± SD *
Fall 2011	*Abramis brama*	0.00103 ± 0.00027 (*n* = 2)
	*Perca fluviatilis*	0.00008 (*n* = 1)
	*Esox lucius*	0.00046 (*n* = 1)
	*Sander lucioperca*	0.00127 ± 0.00054 (*n* = 3)
	*Rutilus rutilus*	0.00018 (*n* = 1)
Spring 2012	*Abramis brama*	0.00200 ± 0.00173 (*n* = 7)
	*Perca fluviatilis*	0.00159 ± 0.00152 (*n* = 3)
	*Esox lucius*	0.00026 (*n* = 1)
	*Sander lucioperca*	0.00642 ± 0.00253 (*n* = 3)

* Mean BMAA concentrations in quantifiable samples of fish muscle tissue ± Standard deviation, *n* = biological replicates.

All kidney and liver samples tested negative for BMAA. Inconclusive BMAA peaks were detected in a few liver samples from *Abramis brama* and *Perca fluviatilis* caught in fall 2011; however, the peaks were too low in intensity to be taken to indicate positive samples. This could be dependent on the matrix effect, since liver is a major site of lipid storage [31,32], and not because the samples were truly BMAA negative. The BMAA isomer DAB was detected in a few kidney samples and, along with BMAA, in almost all positive samples (data not shown).

From the six fish species (*i.e.*, *Tinca tinca*, *Lota lota*, *Salmo trutta trutta*, *Gymnocephalus cernua*, *Scardinius erythrophthalmus*, and *Anguilla anguilla*) caught exclusively in spring 2012, only individuals of *G. cernua*, *T. tinca*, and *A. anguilla* contained quantifiable peaks of BMAA in brain and muscle tissues (Table 4). *G. cernua* was the species with a higher percentage of positive results.

The Spearman’s rank test indicated no significant correlation between the BMAA concentration in brain and muscle tissues (*p* = 0.5026).

**Table 4:** BMAA concentrations in quantifiable brain and muscle samples from the six fish species collected exclusively in spring 2012 in Lake Finjasjön: *Gymnocephalus cernua* (Ruffe), *n* = 15; *Tinca tinca* (Tench), *n* = 15; and *Anguilla anguilla* (Eel), *n* = 15.

Tissue	Species	BMAA, µg·g^−1^ DW ± SD *
Brain	*Gymnocephalus cernua*	0.00864 ± 0.00476 (*n* = 3)
	*Tinca tinca*	0.00141 (*n* = 1)
	*Anguilla anguilla*	0.02202 ± 0.00884 (*n* = 2)
Muscle	*Gymnocephalus cernua*	0.00320 ± 0.00329 (*n* = 4)
	*Tinca tinca*	0.00561 (*n* = 1)

* Mean BMAA concentrations in quantifiable samples of fish tissues ± Standard deviation, *n* = biological replicates.

A suitable linear model explaining the distribution of BMAA concentration in the fish brain contains the fish total weight variable (*p* = 0.0082) and the fish species variable (*p* = 0.0268). However, due to the high amount of negative BMAA samples (*i.e.*, zeros) this model can only explain 9.13% of the variance. Regarding the fish total weight variable, this model predicts that in a group of individuals of the same species, the heaviest one has a higher chance of containing higher amounts of BMAA in its brain.

The distribution of BMAA in the brain samples of the species *E. lucius* (*p* = 0.00063) and *S. lucioperca* (*p* = 0.00494) differs significantly from the distribution observed in *A. brama*. *R. rutilus* and *P. fluviatilis* do not display any significant difference from *A. brama*. BMAA is therefore more frequently found in *A. brama*, *R. rutilus*, and *P. fluviatilis* than in *E. lucius* and *S. lucioperca*.

**Figure 2:**
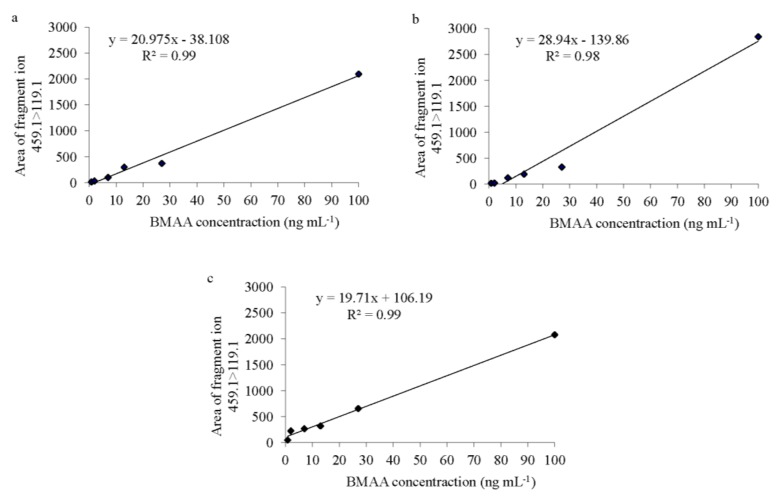
BMAA standard curves of (**a**) *Spirulina* powder, (**b**) Atlantic horse mackerel (*Trachurus trachurus*) brain and (**c**) muscle tissue; with six concentration points (0.8, 2, 7, 13, 27 and 100 ng·mL^−1^) prepared in triplicate over 2.5 mg of protein from each matrix.

The limit of detection (LOD) and limit of quantification (LOQ) were determined based on the signal-to-noise ratio (S/N) of the chromatographic peaks [33]. The LOD was calculated for the diagnostic product ion of BMAA (*i.e.*, *m*/*z* 459.1 > 258.09) and the LOQ for the general BMAA product ion (*i.e.*, *m*/*z* 459.1 > 119.08). The LOD was established when the S/N was higher than 3.0 for the diagnostic product ion of BMAA (*i.e.*, *m*/*z* 459.1 > 258.09). For the general BMAA product ion (*i.e.*, *m*/*z* 459.1 > 119.08), the BMAA peak displayed an S/N between 10 and 30, so the LOQ was described as equal to the LOD. For *Spirulina*, both LOD and LOQ were 0.8 ng·mL^−1^ and the calibration curve had an *r*^2^ = 0.99 (Figure 2a). In the fish samples (in both brain and muscle tissues), both LOD and LOQ were also 0.8 ng∙mL^−1^. The regression lines revealed an *r*^2^ of 0.98 for the brain tissue and 0.99 for the muscle tissue (Figure 2b,c). BMAA was only considered to be present in a sample when a peak had an S/N value higher than 4.0, was observed in both product ions, the BMAA retention time at *m*/*z* 459.1 > 119.08 and *m*/*z* 459.1 > 258.09 chromatograms was 2.85 ± 0.12 min and the ratio between the ions *m*/*z* 459.1 > 119.08 and *m*/*z* 459.1 > 258.09 were 5.5 ± 1.0. Retention time and ions ratio was matrix dependent. Although all BMAA quantifications were performed in the second elute of the solid-phase extraction purified sample, occasionally BMAA peaks were also detected in the first elute.

D_3_-BMAA recovery (*i.e.*, SPE and derivatization) in the blank samples was 54.9 ± 2.0% for the brain and 14.3 ± 3.5% for the muscle tissues. In the environmental contaminated fish samples the D_3_-BMAA losses were equivalent to recovery experiment in the blank samples; 34.3 ± 6.2% for the brain and 10.7 ± 7.5% for the muscle tissues. A matrix effect was also detected, when the D_3_-BMAA signal response in the matrix was compared to the signal in the Borate buffer. Although these values were lower than those presented in previous studies of cyanobacteria and mussels [34,35,36,37], to the best of our knowledge, this is the first time that D_3_-BMAA recovery has been tested in fish brain and muscle tissues.

The average value of mercury in *S. lucioperca* was 0.091 mg·kg^−1^ wet weight (WW) in males and 0.095 in females (Table 5). In *A. brama*, the average value was 0.040 mg·kg^−1^ WW in both males and females. The EU’s maximum permitted mercury concentration in *S. lucioperca* and *A. brama* is 0.5 mg·kg^−1^ WW, meaning that the mercury levels detected in these fish from Lake Finjasjön are much lower than the EU-permitted concentration.

**Table 5:** Weight and mercury concentrations in the muscle tissue of *Abramis brama* (Bream), *n* = 10, and *Sander lucioperca* (Pike-perch), *n* = 10, collected in fall 2011 in Lake Finjasjön.

		*Abramis Brama*		*Sander Lucioperca*	
		Males *n* = 5	Females *n* = 5	Males *n* = 5	Females *n* = 5
**Weight (kg)**	Maximum	1.387	1.045	1.151	1.118
	Minimum	0.832	0.870	0.785	0.875
	Mean	1.178	0.941	0.935	1.048
	Standard deviation	0.216	0.082	0.169	0.098
**Mercury concentration (mg·kg^−1^ WW)**	Maximum	0.044	0.051	0.100	0.120
	Minimum	0.030	0.032	0.081	0.071
	Mean	0.040	0.040	0.091	0.095
	Standard deviation	0.006	0.008	0.007	0.019

## 3. Discussion

The water samples collected in Lake Finjasjön contained BMAA in three of four samples. This reveals a BMAA exposure route to fish at higher trophic levels inhabiting both pelagic and benthic water masses—thereby sustaining BMAA biomagnification in various niches.

The cyanobacteria *Aphanizomenon klebahnii*, previous established as a BMAA producer [11], was the potential BMAA source in Lake Finjasjön during our sampling period. Nevertheless, the recent findings of BMAA producers among the diatom and dinoflagellate groups [12,13] raise the question of whether the other phytoplankton species present (*i.e.*, *Ceratium hirundinella*, *Cryptomonas* sp., *Fragilaria crotonensis*, and *Asterionella formosa*) [26] might also be sources of BMAA. The BMAA concentrations detected are equivalent to those found in cyanobacteria collected in the Baltic Sea [23] and in representative diatom species from the Swedish West Coast [12].

A divergent BMAA pattern can be distinguished between the piscivorous and the plankti-benthivorous fish in Lake Finjasjön. The plankti-benthivorous fish, which had the higher frequency of BMAA, are dominated by *A. brama* and *R. rutilus*. Both these cyprinidae species inhabit the same niche, have similar diets [38], and can moreover hybridize with each other [39,40].

*A. brama* generally feed on benthic animals [38,41], whereas *R. rutilus* feed on detritus, zooplankton, plants, and benthic animals [42]. *A. brama* undergo an ontogenetic habitat and diet shift from foraging on zooplankton in the pelagic habitats to feeding on invertebrates in the bottom sediment. The size of the bream, whose diet shifts from pelagic to benthic feeding, is highly variable and can vary between lakes [43,44].

Between 1992 and 1994, 430 tons of *A. brama* and *R. rutilus* were removed from Lake Finjasjön as a food-web manipulation measure to restore the native fauna and flora composition. After reducing the population of large-sized *A. brama*, a shift was registered in the feeding habits of *A. brama* and *R. rutilus* in earlier life stages, with an increase in feeding on benthic resources [43]. Since 1992 to 2007 these large-scale reductions of *A. brama* and *R. rutilus* were performed, intermittently, by trawling. Since 2010 and to date (2014), *A. brama* and *R. rutilus* have been removed annually, by fyke-netting during the spring spawning and in the fall by ring-seining. The relative proportions of piscivorous and plankti-benthivorous fish have varied since these mass removals started. In 2012, when this study was performed, a test fishery identified balance in the fish community, which comprised equal proportions of piscivorous and plankti-benthivorous fish.

It is not unexpected to observe that *A. brama* individuals have higher concentrations of BMAA given that they continue to feed on benthic prey, where BMAA exposure is anticipated, throughout their various size classes, while older *R. rutilus* move into open-water to feed [43], potentially reducing exposure later in life. Thus, older individuals are most likely to have higher concentrations of BMAA and this difference will be more profound in fish species that have more uniform BMAA exposure as they grow.

In the next trophic level, the pelagic and piscivorous fish species are *E. lucius* and *S. lucioperca*. Some of the BMAA-positive brain samples representing these species have BMAA concentrations higher than approximately 75% of *A. brama* and *R. rutilus* individuals but below the maximum concentrations found in *A. brama*. Therefore, BMAA can bioaccumulate along the fish trophic chain in Lake Finjajön. In accordance with earlier observations in the Baltic Sea [23], south Florida coastal waters [28,29], and Lake Taihu [30], in Lake Finjasjön the presence of BMAA is more frequent in bottom-feeding than pelagic organisms. This again suggests that cyanobacteria and/or other phytoplankton (*i.e.*, diatoms and dinoflagellates) may descend in the water column during the bloom decline, and/or that benthic phytoplankton have higher BMAA concentrations than do the surface blooms of phytoplankton. BMAA was absent from the brain tissue of *S. lucioperca* collected in the Baltic Sea [23]; however, the brain tissue of *S. lucioperca* from Lake Finjasjön contains quantifiable and relatively high amounts of BMAA. This discrepancy may be due to the large array of prey available to pelagic fish in the Baltic Sea and the large dimensions of the Baltic Sea compared with the confined Lake Finjasjön, where *S. lucioperca* may not be able to avoid ingesting BMAA-contaminated prey.

*P. fluviatilis* is a strictly carnivorous fish that undergoes ontogenetic diet shifts from zooplanktivorous, benthivorous, to piscivorous stages [45] and interacts competitively with *R. rutilus* [46,47,48]. In Lake Finjasjön, small *P. fluviatilis* (<80 mm) individuals consume 78% of the zooplankton and 22% of the benthic prey, while the larger individuals (>180 mm) feed almost exclusively on fish [43].

Two of the highest BMAA concentrations were registered in brain samples of *P. fluviatilis* females of approximately 200 g (theoretically large individuals). These BMAA concentrations probably represent the cumulative effect of earlier zooplankton and benthic feeding together with later feeding on BMAA-contaminated prey fish. The similar frequency of BMAA-containing *P. fluviatilis* individuals and BMAA-containing *A. brama* and *R. rutilus* individuals is possibly related to their common benthivorous diet.

Along with the species, the total weight of the individual was statistically significant in relation to BMAA concentrations, the heavier, and likely older, individuals of the same species containing higher BMAA concentrations. Hence, in a system in which BMAA is continuously bioavailable, an organism will bioaccumulate BMAA throughout its life, so older individuals will contain higher concentrations of BMAA.

The influence of organism weight on BMAA quantity can also explain the recorded variances according to gender and season. Females may have higher growth rates [49] and are often larger than males [50,51,52]. As females therefore may consume larger amounts of potentially BMAA-contaminated prey, this may explain the greater amount of BMAA in their brain tissue. As most individuals caught in spring were females, this season also yielded higher concentrations of BMAA.

However, if only the frequency, and not the concentration, of BMAA is taken into account, there is a slightly higher likelihood of BMAA being found in male individuals and in fish captured in fall. This is understandable considering that most fish species, females in particular, tend to reduce their food intake during sexual maturation and throughout the spawning season [53,54].

Overall, BMAA is found less frequently and at lower concentrations in fish muscle tissue than in brain tissue, though *A. brama* is still the fish species with higher percentage of individuals containing BMAA. As suggested earlier, this must be related to this species’ almost exclusively benthivorous diet. The heterogeneous distribution of BMAA between fish tissues was also observed in the Baltic Sea study and was suggested to be related to BMAA’s tendency for protein association [23]. In the present study, however, approximately four times less BMAA was recovered from muscle than from brain tissue. Also the BMAA concentration in brain and muscle tissues were not significant correlated, therefore no major conclusions can be drawn and this issue requires further investigation.

Of the six fish species collected exclusively in spring, only *G. cernua* and *T. tinca* contained BMAA in both brain and muscle tissues, while *A. anguilla* contained BMAA in only the brain tissue. Based on previous results, the bioaccumulation of BMAA in *G. cernua* and *T. tinca* was predictable, because both species feed mostly on benthic food resources [55,56,57] and typically live above soft sediments where they presumably also forage [58]. Another important factor is that the *G. cernua* population tends to increase with increased eutrophication [57]. *A. anguilla*, another bottom-dwelling fish, feeds on the whole aquatic fauna, depending on prey size and availability [59]. Although no definitive conclusion can be drawn based on just two brain samples of *A. anguilla*, these samples did contain some of the highest BMAA concentrations found in the whole study.

The overall concentrations of BMAA in fish tissues from Lake Finjasjön were similar to those in fish tissues from the Baltic Sea [23], though lower than those found in fish from south Florida coastal waters *i.e.*, 58 ± 41 µg·g^−1^ WW in muscle, 588 ± 81 µg·g^−1^ WW in liver, and 1450 ± 687 µg·g^−1^ WW in kidney [29] or in fish from Lake Taihu in China, where the values of BMAA in fish muscle range from 0.07 ± 0.021 to 35.91 ± 13.40 µg·g^−1^ DW [30]. This variance might be due to biological factors, such as Swedish aquatic systems being less affected by BMAA, though a more likely explanation is methodological differences between studies.

An excessive protein-to-derivative ratio during derivatization has earlier been suggested [60] to be the main cause for the low BMAA concentrations detected in the Baltic Sea [23]—consequently this criticism may be applied to the present study since the same extraction method has been used. To exclude this possibility, D_3_-BMAA was added prior to the SPE step to determine the BMAA recoveries from the SPE step as well as the derivatization (all according to suggestions by Cohen [60]). Additionally the same stable isotope was added to the quantified Finjasjön fish samples. Both tests reveal a relatively high D_3_-BMAA loss in brain and muscle fish tissues. Therefore, the BMAA concentrations presented are a fraction of its actual amount. Nevertheless as this fraction percentage is now known, it will allow a direct comparison of BMAA results across samples and research groups. In addition, during the development of the extraction method [61] each step of the procedure was optimized and examined and it was then concluded that the method requires protein concentrations no lower than 2.5 mg due to sample losses during the SPE cleanup. Thus, we conclude that low BMAA concentrations are most probably not due to high protein-to-derivative ratios. The detection of both BMAA and mercury in the muscle tissue of *A. brama* and *S. lucioperca* is notable, as BMAA at non-toxic concentrations potentiates mercury neurotoxicity [27]; the synergetic neurotoxicity of BMAA and mercury may therefore threaten human health despite mercury concentrations being below the EU-permitted level.

Taken together, the data presented here indicate the widespread natural occurrence of BMAA in Lake Finjasjön. This finding, together with BMAA and mercury co-occurrence, raises questions as to the current potential public health risk, since the fish species analyzed are frequently caught and consumed by the surrounding population in the city of Hässleholm. It is therefore urgent to understand which phytoplankton species produce BMAA in Swedish limnic systems and to verify the synergistic effects of BMAA with mercury and other toxins and metals present in the relevant fish species, in order to understand the BMAA bioaccumulation pattern in the trophic chain and better protect consumers.

## 4. Experimental Section

### 4.1. Sampling and Sampling Area Description

The lake used as a eutrophicated model was the shallow Lake Finjasjön (56°08′ N, 13°42′ E) located in southern Sweden near the city of Hässleholm. Lake Finjasjön, at an elevation of 43.2 m above sea level, has a surface area of 10.4 km^2^, average and maximum depths of 3.8 and 12.2 m, respectively, a global retention time of six months, and a catchment area of 260 km^2^. The lake’s surface water temperature averages 19.8 °C in the summer (June–August), reaching a maximum of 22.7 °C [26].

Four water samples were collected from Lake Finjasjön in April 2012. 500 mL water bottles were submerged and filled with surface water. Fish species, selected according to their position in the trophic chain and habitat, were collected from Lake Finjasjön throughout two seasons, *i.e.*, fall (September and October) 2011 and spring (April) 2012. The species were: *Abramis brama* (Bream) *n* = 32, *Perca fluviatilis* (Perch) *n* = 29, *Esox lucius* (Pike) *n* = 22, *Sander lucioperca* (Pike-perch) *n* = 29, and *Rutilus rutilus* (Roach) *n* = 24. In spring 2012, additional fish species were collected: *Tinca tinca* (Tench) *n* = 15, *Lota lota* (Burbot) *n* = 6, *Salmo trutta trutta* (Trout) *n* = 6, *Gymnocephalus cernua* (Ruffe) *n* = 15, *Scardinius erythrophthalmus* (Common Rudd) *n* = 10, and *Anguilla anguilla* (Eel) *n* = 15. Fish specimens were immediately frozen (−20 °C) and transported to the laboratory.

### 4.2. Sample Preparation

Water samples were lyophilized using a CoolSafe freeze-dryer (SCANVAC, Stockholm, Sweden) at −110 °C and stored at −20 °C until BMAA extraction.

Frozen fish samples were thawed at room temperature and then weighed and dissected into brain, muscle, liver, and kidney tissue. Fish tissue samples were ground finely and frozen at −20 °C until use. The fish gender was also registered.

### 4.3. BMAA Sample Extraction

BMAA was extracted from water and fish tissue samples (*i.e.*, brain, muscle, liver, and kidney) according to the procedure of Spacil, *et al.* [61] with minor alterations. Samples were dissolved in 80% methanol (80/20 methanol/water, v/v). Cell lysis was ensured by subjecting the cell suspension to three freeze/thaw cycles in liquid nitrogen, followed by sonication (Sonopuls, Model HD 2070; Bandelin Electronic, Berlin, Germany) for three 45-s cycles at 70% intensity. To minimize protein degradation, all samples were kept in an ice-water bath during sonication and allowed to cool between each sonication cycle. Protein concentration was determined after cell lysis using Bio-Rad RC/DC kit (Bio-Rad, Sundbyberg, Sweden). Samples were hydrolyzed in 6 mmol·L^−1^ HCl for 20 h at 110 °C and then lyophilized using a freeze-dryer at −110 °C. Lyophilized samples were reconstituted with 20 mmol·L^−1^ HCl, and 2.5 mg of protein from each sample was then purified using solid-phase extraction (SPE) (Isolute HCX-3, 100 mg; Biotage, Uppsala, Sweden). Prior to equilibration 1 mL of MeOH was passed through the SPE column. The SPE column was then equilibrated by two consecutive additions of 1 mL 0.1% formic acid. Next, 0.2 mL of sample (*i.e.*, 2.5 mg of protein) was loaded together with 0.1% formic acid (0.8 mL). The SPE column was washed with 1 mL 0.1% formic acid and the first elute (1 mL of 0.1% formic acid in 25% MeOH) was discarded. Then the second elute (1 mL of 2% NH_4_^+^ in MeOH) from each sample was lyophilized using a miVac centrifugal vacuum concentrator (Genevac, Ipswich, UK) connected to a freeze-dryer and stored at −20 °C.

Lyophilized samples were then reconstituted with 20 µL of 20 mmol·L^−1^ HCl solution, transferred to glass vials (Waters, Milford, MA, USA) for LC analysis, and derivatized with AccQ-Tag using a WAT052880 AccQ-Tag kit (Waters), *i.e.*, 70 μL of borate buffer and 30 μL of AQC. Ultra performance liquid chromatography tandem mass spectrometry (UPLC-MS/MS) analysis was performed within 2–10 h of samples derivatization.

### 4.4. Ultra Performance Liquid Chromatography Tandem Mass Spectrometry

Analysis was performed using an Acquity UPLC coupled to a Xevo-TQ-MS (Waters). The separation was performed with an AccQ-Tag Ultra C18 column (100 mm × 2.1 mm, 1.7 µm particle size; Waters) using a binary pump: Eluent A was 0.01% formic acid in 0.05% ammonia in water; eluent B was 0.01% formic acid in methanol. The gradient used was as follows: 0.1% B, 0.54 min; 45% B, 4.00 min; 100% B, 4.10 min; and 0.1% B, 4.70–6.00 min. The flow was diverted to the mass spectrometer between 1.2 and 3.5 min to minimize contamination of the interface; otherwise it was diverted to waste.

Ionization was performed in positive ion mode and the mass analyzer was run in selected reaction monitoring (SRM) scan mode using the following transitions to distinguish BMAA from its isomers *N*-(2-aminoethyl) glycine (AEG) and 4-diaminobutyric acid (DAB): general to all three analytes, 459.1 > 119.1; DAB diagnostic fragment, 459.1 > 188.1; AEG diagnostic fragment, 459.1 > 214.1; BMAA diagnostic fragment, 459.1 > 258.1 and D_3_-BMAA fragment, 462.20 > 122.1. To further ensure positive identification of BMAA, the retention time and fragmentation ratio of fragments 119.1/258.1 were controlled.

All settings were optimized for BMAA detection as follows: cone voltage, 30 V; source temperature, 150 °C; desolvation temperature, 550 °C, cone gas flow 20 L∙h^-1^, desolvation gas flow, 1000 L∙h^-1^, collision gas flow, 0.15 mL∙min^-^1, collision energy, 26 V. MassLynx V4.1 software (Waters) was used to analyze the acquired chromatographic data. BMAA was identified using a highly selective UPLC-MS/MS method that distinguishes BMAA from its isomers AEG and DAB in biological samples.

### 4.5. Quantification Method and Recovery

*Spirulina* powder (Go for Life AB, Stockholm, Sweden) previously determined to contain no detectable amounts of BMAA was selected as a negative control and used as matrix when water samples were quantified. The standard curve has six concentration points (0.8, 2, 7, 13, 27 and 100 ng·mL^−1^) prepared in triplicate over 2.5 mg of *Spirulina* powder protein.

Atlantic horse mackerel (*Trachurus trachurus*) collected in the Atlantic Ocean in April 2012 was dissected and extracted using the same procedure as used for all fish samples. After proving to be negative for BMAA, brain and muscle tissues were used as a negative control and as matrix for brain and muscle tissue quantification. Both standard curves have six concentration points (0.8, 2, 7, 13, 27 and 100 ng·mL^−1^) prepared in triplicate over 2.5 mg of protein of each matrix. All standards were added to the blank matrices just before derivatization.

To test the BMAA recovery, twelve replicates of *T. trachurus* (six brain and six muscle), were prepared. To three samples each of brain and muscle tissue, a fixed amount of D_3_-BMAA internal standard (0.8 ng) was added before solid-phase extraction over 2.5 mg of protein. D_3_-BMAA was added to the other six samples just after the sample preparation but before derivatization.

In addition, to predict BMAA losses, the same fixed amount of D_3_-BMAA was added to every quantified sample prepared during the study before solid-phase extraction.

### 4.6. Statistical Analysis

To ensure accurate statistical analysis, only data for the fish species collected in both seasons were used, consequently the sample size of the analyzed fish species was analogous.

To detect any significant (*p* < 0.05) influence of the variables season of collection, fish gender, total weight, and species on the response variable (*i.e.*, BMAA concentration in brain tissue) distribution, several parametric linear models were executed after the transformation of numerical variables and achievement of Gaussian distributions. Anova-Chi-Square tests were also performed to confirm linear model results.

Although the distribution of BMAA concentrations in brain satisfied the parametric test assumptions, the distribution of BMAA concentrations in the muscle tissue did not. Therefore the non-parametric Spearman’s rank correlation coefficient was used to test whether the BMAA concentrations in brain and muscle tissues were correlated (*p* < 0.05).

Statistical analysis was carried out on R Statistical Software (Foundation for Statistical Computing, Vienna, Austria).

### 4.7. Mercury Analysis

Muscle samples of *Sander lucioperca* (Pike-perch) and *Abramis brama* (Bream) collected from Lake Finjasjön in fall 2011 were tested for mercury. The individuals weighed approximately 1 kg each; 10 individuals of each fish species were selected, 50% of which were males and 50% females.

Total mercury was analyzed using the SS-EN 16277:2012 method [62], which is developed by the Swedish Standards Institute. The method is entitled Determination of mercury by cold-vapor atomic absorption spectrometry (CVAAS) after microwave pressure digestion (extraction with 65% nitric acid and 30% hydrogen peroxide).The analyses were performed at the laboratory of Eurofins Environment Sweden AB, Lidköping, Sweden.
